# ﻿A new species of *Achalinus* Peters, 1869 (Squamata, Xenodermidae) from Hunan Province, China

**DOI:** 10.3897/zookeys.1166.103055

**Published:** 2023-06-13

**Authors:** Shun Ma, Sheng-Chao Shi, Sun-Jun Xiang, Fu Shu, Jian-Ping Jiang

**Affiliations:** 1 Chengdu Institute of Biology, Chinese Academy of Sciences, Chengdu 610041, China Chengdu Institute of Biology, Chinese Academy of Sciences Chengdu China; 2 University of Chinese Academy of Sciences, Beijing 100049, China University of Chinese Academy of Sciences Beijing China; 3 College of Biological and Food Engineering, Huaihua University, Huaihua 418000, China Huaihua University Huaihua China; 4 Central South Academy of Inventory and Planning of NFGA, Changsha 410014, China Central South Academy of Inventory and Planning of NFGA Changsha China

**Keywords:** *Achalinushunanensis* sp. nov., morphology, phylogeny, divergence time, taxonomy

## Abstract

A new species, *Achalinushunanensis***sp. nov.**, is described from middle and western Hunan Province based on the results of molecular systematics and morphological characters. It diverges from known congeners by a significant genetic divergence (*p*-distance 3.2%–16.9% based on CO1 mitochondrial gene), and it can be distinguished from all known congeners by the following morphological characters: (1) all dorsal scales strongly keeled, 23 rows throughout the body, the outmost one strongly keeled and enlarged; (2) tail relatively short, TaL/TL 0.221 ~ 0.225; (3) maxillary teeth 23; (4) the suture between internasals 2 × as long as that between prefrontals; (5) loreal one, subrectangular, LorH/LorL 0.62 ~ 0.70; (6) supralabials 6, the 4^th^ and 5^th^ touch the eye; (7) the two anterior temporals in contact with eye; (8) ventrals 163–165, subcaudals 69–72, not paired. This raises the number of known species of *Achalinus* to 24.

## ﻿Introduction

The odd-scaled snake genus *Achalinus* Peters, 1869, which is widely distributed in northern Vietnam, China, and Japan, is a group of small to medium-sized, nocturnal, fossorial, and non-venomous snakes (Zhao et al. 1998; Zhao 2006). It is the most diverse genus of six known genera of the family Xenodermidae: 23 species in *Achalinus*; two species in *Fimbrios* Smith, 1921; two species in *Parafimbrios* Teynié, David, Lottier, Le, Vidal & Nguyen, 2015; one species in *Paraxenodermus* Deepak, Lalronunga, Lalhmingliani, Das, Narayanan, Das & Gower, 2021; two species in *Stoliczkia* Jerdon, 1870; one species in *Xenodermus* Reinhardt, 1836 (Ha et al. 2022; Yang et al. 2022; Zhang et al. 2023). Among *Achalinus* species, more than half (14/23) have been described since 2019 (Wang et al. 2019; Ziegler et al. 2019; Li et al. 2020; Luu et al. 2020; Miller et al. 2020; Hou et al. 2021; Huang et al. 2021; Li et al. 2021; Ha et al. 2022; Yang et al. 2022; Zhang et al. 2023). Besides, previous studies revealed that *A.ater* Bourret, 1937, *A.formosanus* Boulenger, 1908, *A.huangjietangi* Huang, Peng & Huang, 2021, *A.niger* Maki, 1931, *A.rufescens* Boulenger, 1888, and *A.spinalis* Peters, 1869 required further studied, due to their distinct morphology variation, deep intraspecific divergence, or non-monophyletic relationship (Zhao et al. 1998; Miller et al. 2020; Huang et al. 2021; Zhang et al. 2023). However, because of poor sampling, morphological and molecular information of *Achalinus* were lacking, leading to unresolved taxonomic problems and poorly known distribution patterns. Therefore, the diversity of this genus is underestimated and further research is required.

**Figure 1.**
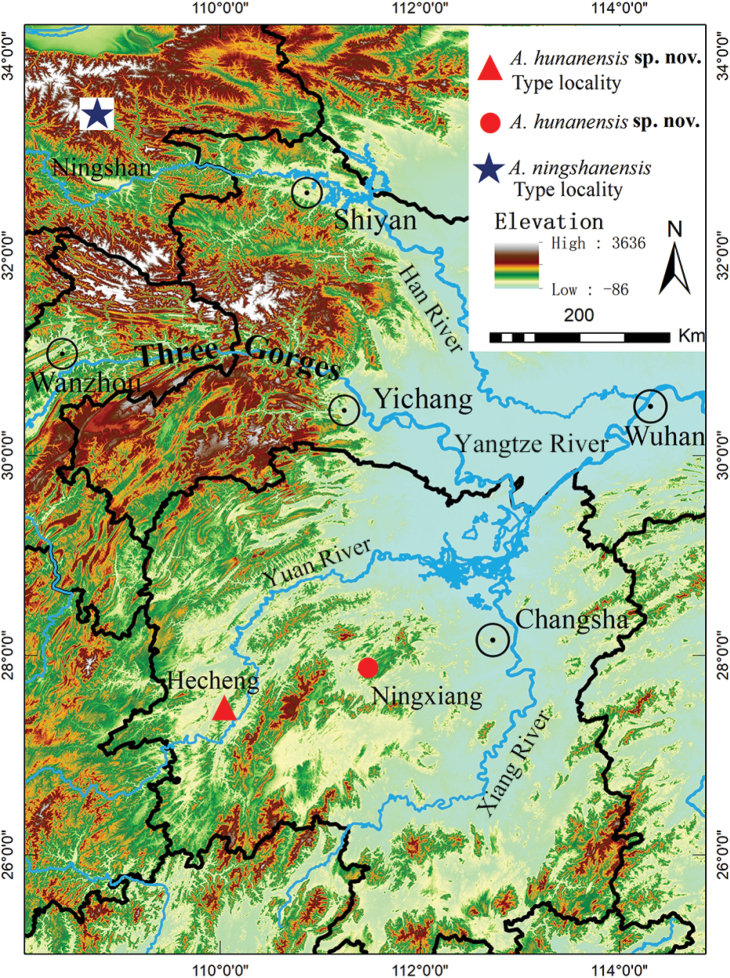
Distribution of *Achalinushunanensis* sp. nov. and its sister taxon *A.ningshanensis*. Blue pentacle: the type locality of *A.ningshanensis*: Xunyangba, Ningshan County, Shaanxi Province, China. Red triangle: the type locality of *A.hunanensis* sp. nov. (CIB 119039): Hecheng District, Huaihua City, Hunan Province, China. Red circle: *A.hunanensis* sp. nov. (CIB 119040): Wazizhai, Ningxiang County, Changsha City, Hunan Province, China.

During our field work, two specimens were collected from Hunan Province, China (Fig. 1). They could be assigned to *Achalinus* by body small, slender, and cylindrical; whole body scales strongly keeled, lanceolate-shaped, and metallic luster; and preocular and postocular absent, loreal and temporals contacting the eyes directly. However, further morphological and molecular analyses revealed that these specimens comprise a separate taxon from other known species. Thus, we described them as a new species herein.

## ﻿Materials and methods

### ﻿Molecular phylogenetic analyses

Two specimens of the genus *Achalinus* were collected in Hunan Province, China: CIB 119039 was collected in Huaihua City, and CIB 119040 was collected in Ningxiang County, and they were deposited in Chengdu Institute of Biology (CIB) of Chinese Academy of Sciences (CAS). Genomic DNA were extracted from preserved muscle tissues of them using QIAamp DNA Mini Kit (QIAGEN, Changsheng Biotechnology Co. Ltd). A fragment of the mitochondrial cytochrome c oxidase subunit 1 (CO1) was amplified using the primer pairs dglco and dghco (Meyer et al. 2005). The polymerase chain reaction (PCR) was performed in 25 μL of reactant with the following cycling conditions: 95 °C for 4 min; 35 cycles of denaturing at 95 °C for 30 s, annealing at 48 °C for 30 s, and extending at 72 °C for 60 s; and a final extending step of 72 °C for 10 min (Wang et al. 2019). PCR products were sequenced by Beijing Qingke New Industry Biotechnology Co., Ltd.

For phylogenetic analysis, 25 sequences (Table 1) were selected, among which 23 sequences (NO. 3–25) were obtained from National Center for Biotechnology Information (NCBI) including 22 sequences from 19 species of *Achalinus* and three sequences of *Fimbriosklossi* Smith, 1921, *Parafimbrioslao* Teynié, David, Lottier, Le, Vidal & Nguyen, 2015 and *Xenodermusjavanicus* Reinhardt, 1836, which were used as outgroups (Luu et al. 2020; Ha et al. 2022).

**Table 1.** Localities, voucher information, GenBank numbers, and references for all samples used in this study.

NO.	Species	Locality	Voucher	CO1 GenBank No.	References
1	*A.hunanensis* sp. nov.	Huaihua, Hunan, China	CIB 119039	OQ848425	This study
2	*A.hunanensis* sp. nov.	Ningxiang, Hunan, China	CIB 119040	OQ848426	This study
3	*A.ningshanensis*	Ningshan, Shaanxi, China	ANU 20220006	ON548422	Yang et al. 2022
4	*A.ningshanensis*	Ningshan, Shaanxi, China	ANU 20220007	ON548423	Yang et al. 2022
5	*A.ater*	Huaping Nature Reserve, Guangxi, China	SYS r00852	MN380334	Wang et al. 2019
6	*A.dehuaensis*	Dehua, Fujian, China	YBU 13013	MZ442662	Li et al. 2021
7	*A.emilyae*	Hoanh Bo, Quang Ninh, Vietnam	IEBR 4465	MK330857	Ziegler et al. 2019
8	*A.formosanus*	Taiwan, China	RN2002	KU529452	Unpublished
9	*A.huangjietangi*	Huangshan, Anhui, China	HSR18030	MT380191	Huang et al. 2021
10	*A.juliani*	Ha Lang, Cao Bang, Vietnam	IEBR A.2018.8	MK330854	Ziegler et al. 2019
11	*A.meiguensis*	Mianyang, Sichuan, China	GP835	MZ442641	Li et al. 2021
12	*A.niger*	Taiwan, China	RN0667	KU529433	Unpublished
13	*A.panzhihuaensis*	Yanbian, Sichuan, China	KIZ 040189	MW664862	Hou et al. 2021
14	*A.pingbianensis*	Honghe, Yunnan, China	YBU 18273	MT365521	Li et al. 2020
15	*A.rufescens*	Hongkong, China	SYS r001866	MN380339	Wang et al. 2019
16	*A.spinalis*	Badagong Mountains, Hunan, China	SYS r001327	MN380340	Wang et al. 2019
17	*A.timi*	Thuan Chau, Son La, Vietnam	IEBR A.2018.10	MK330856	Ziegler et al. 2019
18	*A.tranganensis*	Ninh Binh, Vietnam	VNUF R.2018.21	MW023086	Luu et al. 2020
19	*A.vanhoensis*	Van Ho, Son La, Vietnam	VNUF R.2019.13	ON677935	Ha et al. 2022
20	*A.yangdatongi*	Wenshan Nature Reserve, Yunnan, China	KIZ 034327	MW664865	Hou et al. 2021
21	*A.yunkaiensis*	Dawuling Forestry Station, Guangdong, China	SYS r001443	MN380329	Wang et al. 2019
22	*A.zugorum*	Bac Me, Ha Giang, Vietnam	IEBR 4698	MT502775	Miller et al. 2020
23	*Fimbriosklossi*	Quang Ngai, Vietnam	IEBR 3275	KP410744	Teynié et al. 2015
24	*Parafimbrioslao*	Louangphabang, Laos	MNHN 2013.1002	KP410746	Teynié et al. 2015
25	*Xenodermusjavanicus*	Sumatera Barat, Indonesia:	—	KP410747	Teynié et al. 2015

CO1 sequences (681 bp) were input in MEGA11 (Tamura et al. 2021) and aligned by MUSCLE (Edgar 2004), and then the uncorrected pairwise distances (*p*-distance) were calculated in MEGA11. IQ-TREE 1.6.12 was used to conduct the maximum likelihood (ML) analysis (Nguyen et al. 2015) under the best-fit model TN+F+I+G4 selected by Modelfinder according to BIC (Kalyaanamoorthy et al. 2017). Ultrafast Bootstrap Approximation (UFB) node support as assessed by using 5000 ultrafast bootstrap replicates and the UFB ≥ 95 was considered significantly supported (Hoang et al. 2018). The single branch tests were made using SH-like approximate likelihood ratio test (SH-aLRT) via 1000 replicates, and the SH ≥ 80 was also considered supported well (Stephane et al. 2010). For Bayesian inference (BI), the best-fitting model HKY+I+G was selected by jModelTest 2.1.10 identified via BIC (Darriba et al. 2012) on CIPRES (Miller et al. 2010). The Bayesian inference analysis was conducted using MrBayes v. 3.2.1 (Ronquist et al. 2012) under HKY+I+G this model, and four chains run was calculated for 10 million generations, sampled every 1000 with the first 25% of samples discarded as burn-in, resulting in a potential scale reduction factor (PSRF) of ≤ 0.005. Bayesian posterior probabilities (BI) ≥ 0.95 were considered supported well.

We also estimated divergence time by BEAST v. 2.6.7 using CO1 sequences dataset (Bouckaert et al. 2019). Three calibration constraints were used: the divergence time between *Achalinus* and other Xenodermatidae: 38.6 Mya; the divergence time between *Xenodermus* and *Fimbrios* + *Parafimbrios*: 29.67 Mya; and the divergence time between *Fimbrios* and *Parafimbrios*: 17.66 Mya (Kumar et al. 2022). Two independent searches of 20 million generations were conducted, sampling every 1000 iterations with 25% of the initial samples discarded as burn-in. Tracer v. 1.7.2 was used to evaluate estimate ESS for all parameters (Rambaut et al. 2018).

### ﻿Morphological examination

Morphological data of known species of *Achalinus* were obtained from the two newly collected specimens, examination of museum specimens (*A.ater*: *n* = 1; *A.rufescens*: *n* = 1; *A.yunkaiensis*: *n* = 1) (Appendix 1) and many key references (Boulenger 1888, 1893, 1896; Denburgh 1912; Bourret 1935, 1937; Hu and Zhao 1966; Hu et al. 1973; Koshikawa 1982; Zong and Ma 1983; Ota and Toyama 1989; Zhao et al. 1998; Zhao 2006; Wang et al. 2019; Ziegler et al. 2019; Li et al. 2020; Luu et al. 2020; Miller et al. 2020; Yu et al. 2020; Hou et al. 2021; Huang et al. 2021; Li et al. 2021; Chen et al. 2022; Ha et al. 2022; Xu et al. 2023; Yang et al. 2022; Zhang et al. 2023).

Morphological descriptions followed Zhao (2006) and Yang et al. (2022). Three characters were measured to the nearest 1 mm by Deli Stainless Ruler (No. 8460): snout-vent length (**SVL**), tail length (**TaL**) and total length (**TL**); the other characters were measured to the nearest 0.01 mm by Deli Digital Vernier Caliper (DL91150): head length (**HL**), head width (**HW**), eye horizontal diameter (**ED**), loreal height (**LorH**), loreal length (**LorL**), length of the suture between internasals (**LSBI**), length of the suture between prefrontals (**LSBP**). We counted the following ratios: **TaL/TL**: tail length/total length, **LorH/LorL**: loreal height/loreal length, **LSBI/LSBP**: length of the suture between internasals/length of the suture between prefrontals, **HL/HW**: ratio head length/head width. We also directly compared the length of the sutures between internasals and prefrontals (**LSBI vs. LSBP**).

Scalation features and their abbreviations are as follows: loreals (**Loreal**), supralabials (**SPL**), infralabials (**IFL**), the number of chin shield pairs (**Chins**), the number of infralabials touch the first pair of chin shields (**IFL-1^st^ Chin**), supraoculars (**SPO**), temporals (**TEM**), the number of anterior temporals touch the eye (**aTEM-Eye**) (those head bilateral scale counts were given as left/right), pre-ventral scales (**PrV**), ventral scales (**VEN**), subcaudal (**SC**), entire or divided of the anal (**Anal**), dorsal scale rows (**DSR**) (counted at one-head-length behind the head, at midbody, at one-head-length before the anal). We also counted the number of maxillary teeth (**MT**) under the microscope.

## ﻿Results

### ﻿Molecular systematics

**Figure 2.**
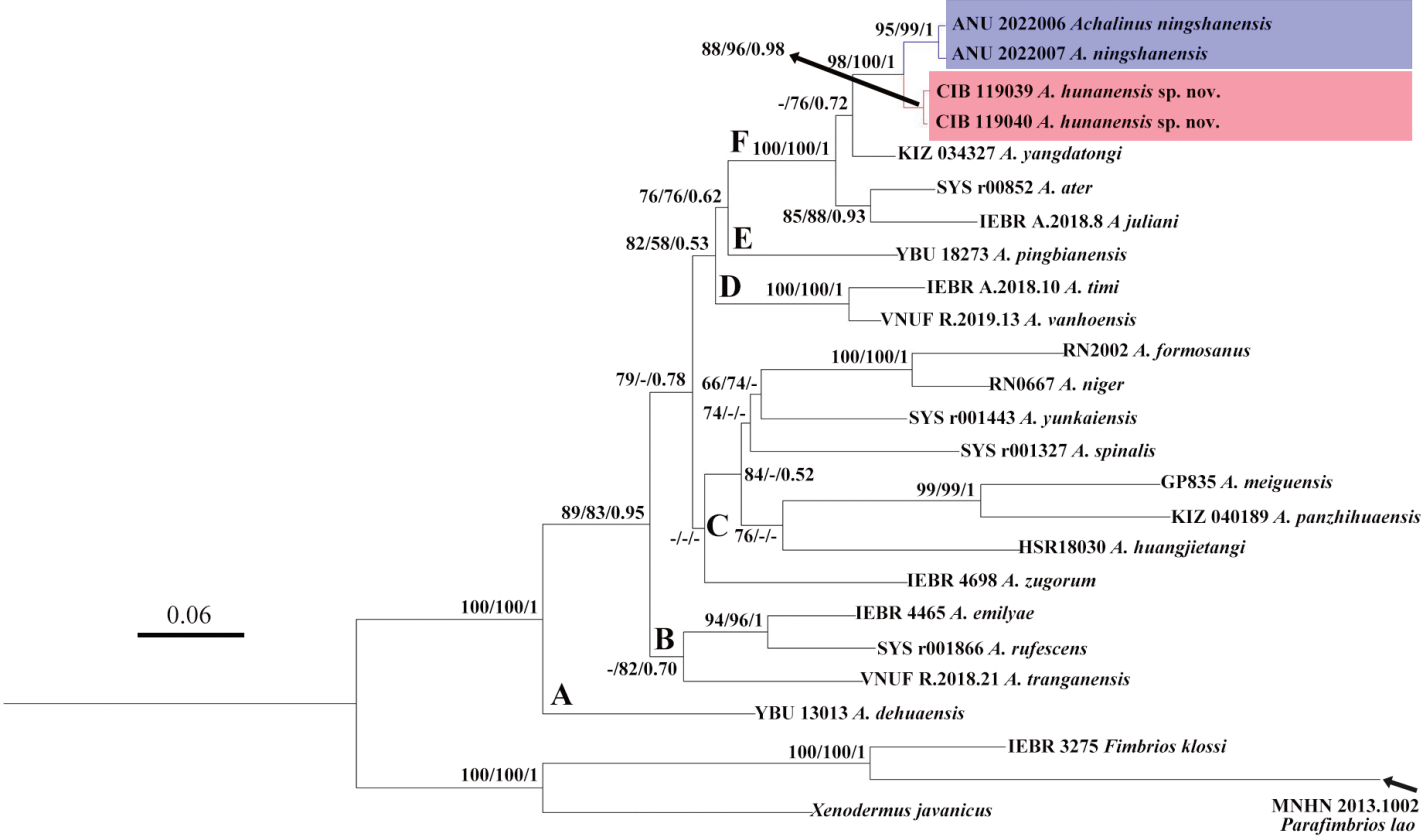
Phylogenetic tree of the genus *Achalinus* inferred from CO1 gene fragment (681 bp) using Maximum Likelihood. The tree nodes present the supporting values: SH-like approximate likelihood ratio test, Ultrafast Bootstrap Approximation and Bayesian posterior probabilities, respectively (SH, %/UFB, %/BI) (the ones lower than 50 are displayed as “-”). *Achalinusningshanensis* is noted in blue and *A.hunanensis* sp. nov. is noted in red.

**Figure 3.**
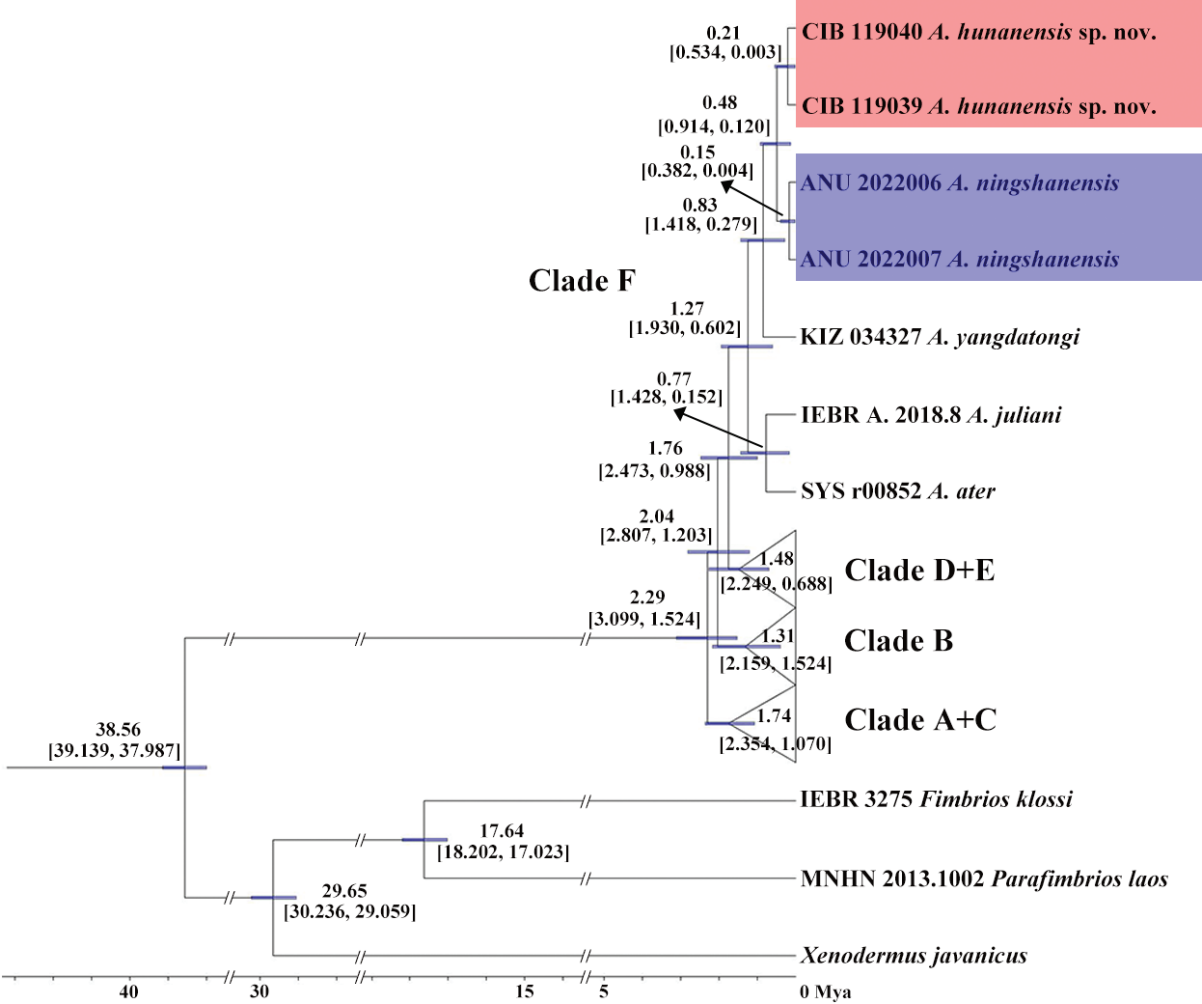
Divergence date estimation (Mya) and 95% HPD (in square bracket) of Clade F. *Achalinusningshanensis* is in blue and *A.hunanensis* sp. nov. is in red.

All *Achalinus* samples cluster in a monophyletic group with high supporting values (SH 100/ UFB 100/ BI 1), and they can be divided into six clades, although the relationships among these clades are still unresolved (Fig. 2). Clade A contains *A.dehuaensis* Li, Wu, Xu. Zhu, Ren, Guo & Dong, 2021; clade B contains *A.emilyae* Ziegler, Nguyen, Pham, Nguyen, Pham, Van Schingen, Nguyen & Le, 2019, *A.rufescens* and *A.tranganensis* Luu, Ziegler, Ha, Lo, Hoang, Ngo, Le, Tran & Nguyen, 2020; clade C *A.zugorum* Miller, Davis, Luong, Do, Pham, Ziegler, Lee, De Queiroz, Reynolds & Nguyen, 2020, *A.huangjietangi*, *A.panzhihuaensis* Hou, Wang, Guo, Chen, Yuan & Che, 2021, *A.meiguensis* Hu & Zhao, 1966, *A.spinalis*, *A yunkaiensis* Wang, Li & Wang, 2019, *A.niger* and *A.formosanus*; Clade D includes *A.timi* Ziegler, Nguyen, Pham, Nguyen, Pham, Van Schingen, Nguyen & Le, 2019 and *A.vanhoensis* Van Ha, Ziegler, Sy, Le, Nguyen & Luu, 2022, and Clade E contains *A.pingbianensis* Li, Yu, Wu, Liao, Tang, Liu & Guo, 2020, respectively. Clade F consists five species with a significantly high nodal support (SH 100/ UFB 100/ BI 1; divergence time: 1.76 Mya, 95% highest posterior density interval (HPD): 2.473 ~ 0.988 Mya), of which, the two specimens newly collected in this work are firstly clustered together as a lineage with well support (SH 88/UFB 96/BI 0.98; divergence time: 0.21 Mya, 95% HPD: 0.534 ~ 0.003 Mya), and then clustered with *A.ningshanensis* Yang, Huang, Jiang, Burbrink, Gong, Yu, Zhang, Huang & Huang, 2022 (SH 98/UFB 100/BI 1; divergence time: 0.48 Mya, 95% HPD: 0.914 ~ 0.120 Mya), forming a sister group of *A.yangdatongi* Hou, Wang, Guo, Chen, Yuan & Che, 2021 diverged at 0.83 Mya (95% HPD: 1.418 ~ 0.279 Mya), and then they form a sister group of the lineage which contains the *A.ater* and *A.juliani* Ziegler, Nguyen, Pham, Nguyen, Pham, Van Schingen, Nguyen & Le, 2019 (divergence time: 1.27 Mya, 95% HPD: 1.930 ~ 0.602 Mya) (Fig. 3).

The genetic distances range from 5.0% (*A.timi* and *A.vanhoensis*) to 18.1% (*A.dehuaensis* and *A.meiguensis*) among the known *Achalinus* species studied in this work (Table 2), of which, within the Clade F, the genetic distances among the four known species range from 5.8% (*A.ningshanensis* and *A.yangdatongi*) to 9.6% (*A.juliani* and *A.ningshanensis*), while the lineage composed by those two newly collected specimens range from 3.2% to 8.8% divergent from congeners.

**Table 2.** Uncorrected *p*-distances (%) among *Achalinus* species inferred from mitochondrial CO1 gene.

	1–2	3–4	5	6	7	8	9	10	11	12	13	14	15	16	17	18	19	20	21	22
1–2 *A.hunanensis* sp. nov.	0.5																			
3–4 *A.ningshanensis*	3.2–3.3	0.7																		
5 *A.ater*	7.1–7.3	7.6–7.7																		
6 *A.dehuaensis*	15.1–15.2	16.3–16.5	16.5																	
7 *A.emilyae*	12.9–13.3	13.5–14.1	11.7	15.7																
8 *A.formosanus*	13.8–13.9	14.8–15.1	14.1	15.7	14.6															
9 *A.huangjietangi*	16.8–16.9	17.2	15.0	16.8	14.8	16.2														
10 *A.juliani*	8.7–8.8	9.1–9.6	7.1	15.2	11.5	13.4	14.8													
11 *A.meiguensis*	16.4	17.0	15.4	18.1	15.4	15.6	15.2	16.8												
12 *A.niger*	13.2–13.3	14.6	13.5	15.7	12.9	9.0	14.6	12.9	13.9											
13 *A.panzhihuaensis*	16.2	17.1–17.4	16.2	15.3	16.6	16.0	15.2	15.5	11.6	14.4										
14 *A.pingbianensis*	11.1–11.2	11.7–12.4	11.8	14.8	13.0	14.5	13.0	12.2	16.8	11.7	14.9									
15 *A.rufescens*	12.1–12.2	12.3–12.7	12.7	14.3	8.0	14.1	14.3	12.3	17.3	12.7	16.0	12.9								
16 *A.spinalis*	14.0–14.3	15.1–15.6	15.2	14.3	13.9	13.9	13.4	13.9	16.0	13.5	15.8	13.3	13.0							
17 *A.timi*	12.1	13.6	13.3	15.8	12.9	14.0	14.8	13.7	15.8	12.0	15.5	12.2	13.9	14.3						
18 *A.tranganensis*	13.7–14.2	14.3–15.2	12.7	14.2	10.6	17.3	13.7	12.3	16.4	14.9	16.4	13.3	11.5	14.6	13.5					
19 *A.vanhoensis*	11.3–11.7	12.1–12.4	13.1	15.8	12.3	14.1	14.8	13.5	15.6	12.6	15.5	10.8	13.8	12.9	5.0	13.3				
20 *A.yangdatongi*	5.1	5.8–5.9	6.2	14.0	12.8	14.4	14.6	7.3	17.1	13.7	15.5	11.3	11.5	14.2	13.1	12.8	11.3			
21 *A.yunkaiensis*	11.7–12.1	13.0–13.7	12.8	14.7	13.1	12.3	12.5	12.5	15.8	12.2	15.7	11.6	13.3	12.0	14.1	13.5	13.6	12.0		
22 *A.zugorum*	11.6–11.9	12.8	13.1	14.3	12.9	13.7	14.4	13.5	15.0	13.4	15.3	10.9	13.5	13.3	13.4	12.5	12.0	12.2	10.9	

The results above indicate that the Hunan samples are close to the species *A.ningshanensis* but consist independent evolution lineage.

### ﻿Morphological systematics

The two specimens of the genus *Achalinus* newly collected from Hunan Province can be easily distinguished from all other known congeners (Tables 3, 4). By having 23-23-23 dorsal scale rows, the two specimens can be distinguished from these species including *A.formosanusformosanus* Boulenger, 1908 (vs. 29-27-25), *A.f.chigirai* Ota & Toyama, 1989 (vs. (25–27)-(25–27)-25), *A.meiguensis* (vs. (21–23)-(19–21)-(19–21)), *A.niger* (vs. 25-25-23), *A.panzhihuaensis* (vs. 23-23-19), *A.timi* (vs. 25-25-23), *A.tranganensis* (vs. 25-23-23), *A.vanhoensis* (vs. 25-23-23), *A.zugorum* (vs. 25-23-23). By having loreal separated from prefrontal, they are different from *A.jinggangensis* and *A.pingbianensis* (vs. loreal fused with prefrontal). By having LSBI vs. LSBP > 1, they differ from *A.dabieshanensis* Zhang, Liu, Huang, Hu, Yu, Sun, Zhang, Wen & Zhang, 2023 (vs. < 1), *A.hainanus* Huang, 1975 (vs. = 1), *A.huangjietangi* (vs. < 1), *A.spinalis* (vs. < 1), *A.werneri* Van Denburgh, 1912 (vs. = 1) and *A.yunkaiensis* (vs. = 1). By having more ventrals (163–165), they can be distinguished from *A.dehuaensis* (vs. 142–154), *A.emilyae* (157–161), and *A.rufescens* (vs. 132–156).

**Table 3.** Main morphological characters of *Achalinushunanensis* sp. nov.

Voucher Number	CIB 119039	CIB 119040
	Holotype	Paratype
**Sex**	Male	Male
**SVL**	255	204
**TaL**	74	58
**TL**	329	262
**TaL/TL**	0.225	0.221
**Loreal**	1/1	1/1
**LorH**	1.03/1.04	0.91/0.93
**LorL**	1.54/1.58	1.46/1.49
**LorH/LorL**	0.70/0.66	0.62/0.62
**LSBI**	1.78	1.52
**LSBP**	0.88	0.76
**LSBI/LSBP**	2.02	2.00
**LSBI vs. LSBP**	> 1	> 1
**HL**	7.91	6.33
**HW**	4.80	3.38
**HL/HW**	1.65	1.87
**ED**	1.42/1.41	1.37/1.36
**MT**	23	23
**SPL**	3-2-1/3-2-1	3-2-1/3-2-1
**IFL**	5/6	5/5
**IFL-1^st^ Chin**	3/4	3/3
**SPO**	1/1	1/1
**TEM**	2+2+4/2+2+4	2+2+4/2+2+4
**aTEM-Eye**	2/2	2/2
**PrV**	2	2
**VEN**	163	165
**SC**	69	72
**Anal**	Entire	Entire
**DSR**	23-23-23	23-23-23

**Table 4.** Morphological characters of *Achalinus* obtained from specimens examined in this study and literatures (Boulenger 1888, 1893, 1896; Denburgh 1912; Bourret 1935, 1937; Hu and Zhao 1966; Hu et al. 1973; Koshikawa 1982; Zong and Ma 1983; Ota and Toyama 1989; Zhao et al. 1998; Zhao 2006; Wang et al. 2019; Ziegler et al. 2019; Li et al. 2020; Luu et al. 2020; Miller et al. 2020; Yu et al. 2020; Hou et al. 2021; Huang et al. 2021; Li et al. 2021; Chen et al. 2022; Ha et al. 2022; Yang et al. 2022; Zhang et al. 2023; Xu et al. 2023). Int. fus.: internasal fused to prefrontal; Pre fus.: prefrontal fused to loreal; PtO: postoculars.

Species	TaL/TL	MT	Int fus.	Pre fus.	LorH/LorL	LSBI vs. LSBP	DSR	PtO	SPL	SPL-Eye	IFL	IFL-1^st^ Chin	TEM	aTEM-Eye	VEN	SC
*A.ater*	0.190 ~ 0.220	–	0	0	0.40	> 1	(21–23)-(21–25)-(21–25)	0	6	4–5	5–6	1–3	2+2+3	2	160–170	47–70
*A.dabieshanensis*	0.168 ~ 0.223	–	0	0	0.73 ~ 0.83	< 1	23-23-23	0	6	4–5	5	1–3	2+2+3(4)	2	141–155	45–55
*A.dehuaensis*	0.206 ~ 0.286	31–33	0	0	–	> 1	23-23-23	0	6	4–5	5	1–3	2+2(3)+3(4)	1–2	142–154	63–81
*A.emilyae*	0.183 ~ 0.203	27–28	0	0	–	> 1	23-23-23	0	6	4–5	5	1–3	2+2+3	1	157–161	56–63
*A.formosanuschigirai*	0.317	14	0	1	–	= 1	(25–27)-(25–27)-25	0	6	4–5	5–6	–	2+2	2	161–167	96–97
*A.f.formosanus*	0.159	17	0	1(usually)	–	= 1	29-27-25	0	6	4–5	6–7	–	2+2	1	158–184	61–83
*A.hainanus*	0.258 ~ 0.266	–	0	0	–	= 1	23-23-23	0	6	4–5	5	1–3	1+2+3(4)	1	165–168	67–69
*A.huangjietangi*	0.152 ~ 0.232	–	0	0	0.70 ~ 0.74	< 1	23-23-23	0	6	4–5	5–6	1–3(4)	2+2+3(4)	2	157–170	40–67
*A.hunanensis* sp. nov.	0.221 ~ 0.225	23	0	0	0.62 ~ 0.70	> 1	23-23-23	0	6	4–5	5–6	1–3(4)	2+2+4	2	163–165	69–72
*A.jinggangensis*	0.174 ~ 0.217	–	0	1	–	> 1	23-23-23	0	6	4–5	6	1–4	2(1)+2+3(4)	2	156–164	51–64
*A.juliani*	0.224 ~ 0.268	28	0	0	–	> 1	25-23-23	0	6(7)	4–5(5–6)	6	1–3(4)	2+2+4	2	163–179	77–91
*A.meiguensis*	0.142 ~ 0.238	17	1	0	–	–	(21–23)-(19–21)-(19–21)	1	6	4–5	6	1–3	2(3)+2(3)	1	146–173	39–60
*A.niger*	0.151 ~ 0.179	–	0	0	0.67	< 1	25-25-23	0	6	4–5	6	1–3(4)	2+2(3)	2	169–185	52–72
*A.ningshanensis*	0.121 ~ 0.161	–	0	0	0.45 ~ 0.58	= 1	23-23-23(21)	0	6	4–5	5	1–2(3)	2+2(3)+3(4)	1–2	159–174	41–46
*A.panzhihuaensis*	0.246	28	1	0	0.67	–	23-23-19	1	6	4–5	6	1–3	2+2+3	1	160	73
*A.pingbianensis*	0.243	–	0	1	–	= 1	23-23-23	0	7	5–6	6	1–3	2+2+3	1	164	56
*A.rufescens*	0.191 ~ 0.276	23	0	0	0.80 ~ 1.00	> 1	23-(23–25)-23	0	6	4–5	5	1–3	2(1)+2+3(4)	1–2	132–156	58–82
*A.spinalis*	0.150 ~ 0.250	16–20	0	0	–	< 1	(23–25)-(23–25)-(23–25)	0	6	4–5	5–6	1–3	2+2(3)	1–2	138–175	48–67
*A.timi*	0.213	27	0	1	–	> 1	25-25-23	0	6	4–5	6	1–3	2+2+3	1	170	72
*A.tranganensis*	0.254(+)	29	0	0	–	> 1	25-23-23	0	6	4–5	6	1–3	2+2+3	2	171	73(+)
*A.werneri*	0.250 ~ 0.300	–	0	0	–	= 1	?-(21–23)-?	0	6	4–5	6	–	2+3(4)	–	157–191	67–98
*A.yangdatongi*	0.180 ~ 0.262	24–26	0	0	0.57	> 1	23-23-23	0	6	4–5	5–6	1–3	2+2/3+2/3	2	155–171	59–76
*A.yunkaiensis*	0.156 ~ 0.203	20–24	0	0	0.49 ~ 0.64	= 1	23-23-23	0	6	4–5	6	1–3(4)	2+2+3(4)	2	150–162	49–56
*A.vanhoensis*	0.264	32	0	1	–	> 1	25-23-23	0	6/7	4–5/5–6	6	1–4	2+2+3	2	176	84
*A.zugorum*	0.229	28	0	1	–	> 1	25-23-23	0	6	4–5	7	1–3	2+2+3	2	173	70

Within the clade F, the two newly collected specimens from Hunan can be identified from *A.ater* by having nostril in the anterior part of the nasal (vs. nostril in the posterior part of the nasal), loreal length ~ 1.5 × than loreal height (vs. loreal length > 2 × than loreal height), and more subcaudals (69–72 vs. 47–70). They are different from *A.juliani* by having different dorsal scale rows (23-23-23 vs. 25-23-23), less maxillary teeth (23 vs. 28), and less subcaudals (69–72 vs. 77–91). They differ from *A.yangdatongi* by having relatively shorter tail length in males (0.221 ~ 0.225 vs. 0.261 ~ 0.262), more ventrals in males (163–165 vs. 155), and fewer subcaudals in males (69–72 vs. 76) fewer maxillary teeth (23 vs. 24–26). They also can be easily distinguished from its sister group *A.ningshanensis* by the following morphological characters: (1) the suture between the internasals 2 × as long as the suture between the prefrontals vs. the suture between the internasals subequal to the suture between the prefrontals; (2) relatively longer tail (TaL/TL: 0.221 ~ 0.225 vs. 0.121 ~ 0.161); (3) more subcaudals: 69–72 vs. 41–46; (4) two chin pairs vs. three chin pairs; (5) relatively narrow and long loreals (0.62 ~ 0.70 vs. 0.45 ~ 0.58) (more details are presented in Table 5).

**Table 5.** Main morphological characters of *Achalinushunanensis* sp. nov. and *A.ningshanensis*.

Species	*Achalinushunanensis* sp. nov.	*A.ningshanensis*
**Sex**	Males (*n* = 2)	Females (*n* = 5)
**SVL**	204–255	334–463
**TaL**	58–74	62–72
**TL**	262–329	398–527
**TaL/TL**	**0.221 ~ 0.225**	**0.121 ~ 0.161**
**Loreal**	1/1	1/1
**LorH/LorL**	0.62 ~ 0.70	0.45 ~ 0.58
**LSBI/LSBP**	**2.00 ~ 2.02**	**0.95 ~ 1.11**
**LSBI vs. LSBP**	**> 1**	= **1**
**HL**	6.33–7.91	11.17–13.72
**HW**	3.38–4.80	4.75–7.48
**HL/HW**	1.65–1.87	1.78–2.67
**MT**	23	—
**SPL**	3-2-1	3-2-1
**IFL**	5–6	5
**Chins**	**2**	**3**
**IFL-1^st^ Chin**	3–4	2–3
**SPO**	1	1
**TEM**	2+2+4	2+2+3/2+2+4/2+3+4
**aTEM-Eye**	2	1–2
**VEN**	163–165	159–174
**SC**	**69–72**	**41–46**
**Anal**	Entire	Entire
**DSR**	23-23-23	23-23-23 (rarely 21)
**References**	This study	Yang et al. 2022

Combined the results of molecular systematics and morphological characters above, the specimens newly collected in this work represent a new species, and we describe it herein.

### ﻿Taxonomic account

#### ﻿ Achalinus hunanensis sp. nov.

Taxon classificationAnimaliaSquamataXenodermidae

E15BDB02-9B49-5278-8688-0412904540A3

https://zoobank.org/05012A7E-84CE-4C6D-A915-51FCF365DFFE

Figs 4 , 5

##### Chresonymy.

*Achalinusater*: Shu et al. 2014; Shen et al. 2014; Gao et al. 2022.

##### Material examined.

**Figure 4.**
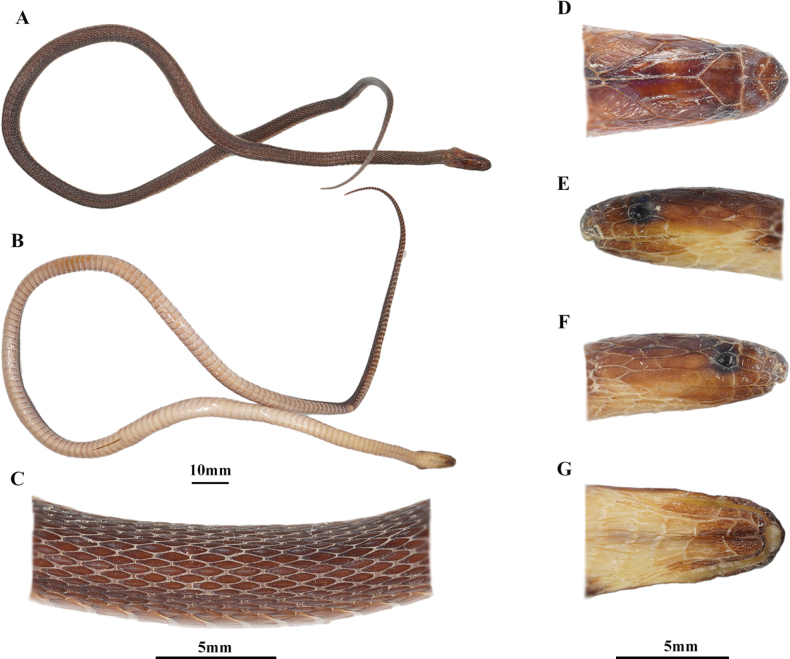
The holotype (CIB 119039, subadult male) of *Achalinushunanensis* sp. nov. **A** dorsolateral view **B** ventral view **C** right side of middle body view **D** dorsal head view **E** left side of head view **F** right side of head view **G** ventral head view. Photographs by SCS.

***Holotype*.**CIB 119039 (Collection No. 20130505001), subadult male (Fig. 4), collected in early May 2013, by Sheng-Chao Shi and Sun-Jun Xiang from Huangyan Village, Hecheng District, Huaihua City, Hunan Province, China (27°28′N, 110°02′E; ca. 880 m a.s.l.).

**Figure 5.**
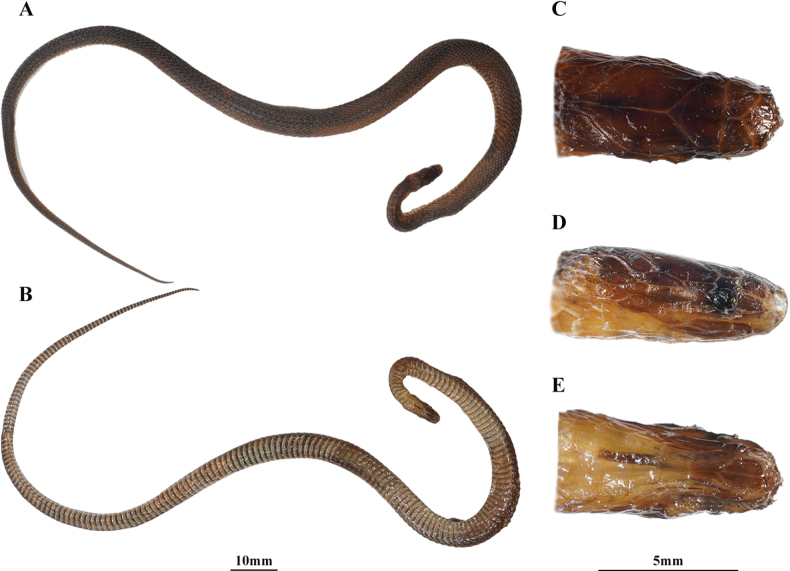
The paratype (CIB 119040, subadult male) of the *Achalinushunanensis* sp. nov. **A** dorsolateral view **B** ventral view **C** dorsal head view **D** right side of head view **E** ventral head view. Photographs by SCS.

***Paratype*.**CIB 119040, subadult male (Fig. 5), collected on 16 June 2022, by Sheng-Qiang Liu from Wazizhai, Ningxiang County, Changsha City, Hunan Province, China (28°00′N, 111°53′E; ca. 1020 m a.s.l.).

##### Etymology.

This new species is named after its known distribution range, which is endemic to Hunan Province. The Chinese name is suggested as “湖南脊蛇” (Hú Nán Jǐ Shé) and the English name “Hunan Odd-scale Snake” or “Hunan Burrowing Snake” is suggested.

##### Diagnosis.

(1) 23 rows of dorsal scales throughout the body, all dorsal scales strongly keeled, and the outmost one strongly keeled and enlarged; (2) tail relatively short, TaL/TL 0.221 ~ 0.225; (3) maxillary teeth 23; (4) the suture between internasals 2 × as long as that between prefrontals; (5) loreal one, subrectangular, LorH/LorL 0.62 ~ 0.70; (6) supralabials six, the 4^th^ and 5^th^ touch the eye; (7) the two anterior temporals in contact with eye; (8) ventrals 163–165, subcaudals 69–72, not paired.

##### Description of holotype.

A subadult male with a total length of 329 mm (SVL 255 mm and TaL 74 mm); tail relatively short, Tal/TL 0.225; body slender, cylindrical; head length (HL) 7.91 mm, head width 4.80 mm, HL/HW 1.65, slightly distinct from neck; eye small, ED 1.42/1.41 mm; maxillary teeth 23, small, equally sized and curved. Rostral small, triangular, only the upper tip visible from above. Length of the suture between the internasals (LSBI 1.78 mm) ~ 2 × as long as length of the suture between the prefrontals (LSBP 0.88 mm). Nostril in the anterior part of the nasal. Loreal one, subrectangular, loreal height (LorH) 1.03/1.04 mm, loreal length (LorL) 0.70/0.66 mm, LorH/LorL 0.62 ~ 0.70. Supraocular one. Frontal one, pentagonal, pointed backwards, much shorter than the parietals. Parietals paired and elongated. No preoculars and postoculars. Temporals 2+2+4, the anterior two contact the eye, the lower anterior temporal much larger, the upper medium temporal much larger, the upper posterior temporal much larger and separated from the other side one by two small scales which contact the parietals. Supralabials 6, 4^th^ and 5^th^ contact the eye, the last one much elongated. One mental. Two chin shields, similar length. Infralabials 5/6, the first one contact with each other after the mental and before the 1^st^ chin shields, 1^st^–3^rd^/1^st^–4^th^ touch the 1^st^ chin shields.

Dorsal scales lanceolate and strongly keeled; 23 rows throughout the body; those of the outmost rows on both sides significantly enlarged and strongly keeled. Ventrals 163, with two preventrals; anal entire; subcaudals 69, not paired.

##### Coloration of holotype.

In life, dorsum dark, slightly metallic, vent black-brown, dark brown near the margin, grey in the margin. A yellowish brown patch on the head occipital. The head ventral anterior part dark brown and posterior part yellowish white (Shu et al. 2014). In preservation, dorsum brown, vent anterior part grey and posterior part light brown. Ventral side of tail brown. The head ventral anterior part brown and posterior part grey.

##### Variations.

Main morphological characters were listed in Table 3. The other sample are very similar to the holotype except that: (1) more ventrals: CIB 119040: 165; (2) more subcaudals: CIB 119040: 72; (3) vent coloration: CIB 119040: dark brown throughout the vent.

##### Distribution and habits.

*Achalinushunanensis* sp. nov. is currently only known from Hunan Province, China: Hecheng District, Huaihua City and Ningxiang County, Changsha City (880–1020 m a.s.l.). The holotype was found at night, near a mountain stream (AT 24 °C, RH 80%) with shrubs under subtropical evergreen broadleaves forest. It was moving from leaf litter to the stream. Earthworms were found at the same place, which we speculated as its prey (Shu et al. 2014).

## ﻿Discussion

Based on molecular evidence, the newly collected *Achalinus* specimens in this study are most closely to *A.ningshanensis* but a genetic differentiation (*p*-distance 3.2%) already exists between these two groups (Fig. 2, Table 2). The newly collected *Achalinus* specimens and its sister group *A.ningshanensis* have separate distribution ranges at south of Yangtze River and north of Yangtze River, respectively, isolated by Three Gorges (Fig. 1). In addition, their estimated divergence time was at 0.48 Mya (Fig. 3), which broadly coincides with the formation of Three Gorges of the Yangtze River (0.30 ~ 0.12 Mya) (Zhang et al. 2018), hindering their communication and driving allopatric speciation. Moreover, combining their distinct morphological differences (Table 5), we describe them as a new species. Currently, 24 *Achalinus* species are reported.

*Achalinusater* was first recorded in Hunan Province only based one specimen (Shen et al. 2014; Shu et al. 2014; Gao et al. 2022). However, this study found that this record was a misidentification and this specimen was designed as the new species holotype. Therefore, *A.ater* recorded on Hunan reptile check list should be transferred as *A.hunanensis*.

Due to the secretive life history and morphological similarities, many cryptic species may be “hidden” within known widely distributed species, such as *A.spinalis*, *A.rufescens*, and *A.ater* (Wang et al. 2019; Yang et al. 2022), and the description of *A.hunanensis* sp. nov. indicated that further study is necessary to conduct by using different *Achalinus* species and geographic populations to revise the mystery snakes and reveal their evolutionary history.

## Supplementary Material

XML Treatment for Achalinus hunanensis

## ﻿Additional information

### Author ORCIDs

Shun Ma https://orcid.org/0009-0003-8611-4550

Sheng-Chao Shi https://orcid.org/0000-0003-2337-6572

Sun-Jun Xiang https://orcid.org/0009-0003-6692-7087

Fu Shu https://orcid.org/0000-0002-6082-8112

Jian-Ping Jiang https://orcid.org/0000-0002-1051-7797

### Data availability

All of the data that support the findings of this study are available in the main text.

## ﻿Appendix 1

**Examined *Achalinus* specimens**

*A.ater* (*n* = 1): China, Guizhou Province, Xingyi County: CIB 63III5243.

*A.rufescens* (*n* = 1): China, Hong Kong: CIB 119042.

*A.yunkaiensis* (*n* = 1): China, Hunan Province, Xinning County: CIB 119041.
